# Dry Treatment of Uterine Disease

**Published:** 1886-05

**Authors:** 


### Dry Treatment of Uterine Disease.

Before the St. Louis Obstetrical and Gynæcological Society, Dr. Engleman has recently advocated a new form of uterine treatment, as opposed to the old methods. After reviewing at length the methods of uterine treatment that have prevailed, and do now prevail among the less advanced of practitioners, and deprecating the introduction of caustics and irritating fluids into the cavities of the cervix and uterus, he proceeds to show how much better results can be obtained, less risks run of cellulitis, etc., with less pain, by a milder, more cleanly and more satisfactory method, known as the "dry treatment."

His plan consists in packing the vagina with medicated absorbent cottons, charged with iodine, iron, alum, or any medicament desired. Often powders, as bismuth, iodoform, etc., are blown upon the cervix and vaginal surface before introducing the cotton. The advantages claimed are, that a larger surface for absorption of the medicament is given, a less intense and irritating treatment, but more prolonged.

Thus, instead of the immediate irritant and caustic action of mopping the uterine cavity with tincture of iodine, by applying iodized cotton, no irritation is produced, no caustic action, but absorption of iodine for a number of hours.

It seems that, in view of the very decided stand that the most experienced gynæcologists have taken against intra-uterine medication, this modified treatment can but meet with favor among the more experienced gynæcologists.

